# Fostering Students’ Sense of School Belonging: Emotional Intelligence and Socio-Ecological Perspectives

**DOI:** 10.3390/jintelligence13090112

**Published:** 2025-09-02

**Authors:** Hatice Turan Bora, Sadegül Akbaba Altun

**Affiliations:** Faculty of Education, Başkent University, Ankara 06790, Türkiye; akbabas@baskent.edu.tr

**Keywords:** sense of school belonging, emotional intelligence theory, socio-ecological theory, student

## Abstract

A strong sense of school belonging is essential for students’ academic achievement, emotional well-being, and overall development. This study explores the role of emotional intelligence and the social environment in shaping students’ sense of belonging. Adopting a basic qualitative approach, this study analyzes teachers’ perspectives on the contributions of students, teachers, parents, counselors, school principals, and the wider society. Qualitative data which were collected through interviews with 49 teachers (37 female, 12 male; years of experience mean is 12) were analyzed through content analysis to identify the main themes. The findings suggest that students’ sense of belonging improves when they actively participate in school life and are aware of the importance of school. Furthermore, students’ sense of belonging improves when teachers offer academic and emotional support, parents engage actively, counselors foster well-being, and school principals provide strong leadership and relationship management. In addition, increased social involvement enhances student belonging. This study offers valuable insights for educators and policymakers in fostering supportive school environments. It highlights the emotional and social processes underlying school belonging and discusses their implications for future research.

## 1. Introduction

Belonging is a basic need for individuals (Strayhorn 2012, p. 19) and a fundamental source of motivation (Baumeister and Leary 1995). As with many social groups, “sense of school belonging (SoSB)” fulfills an individual’s need for support. SoSB includes all formal and informal social interactions that students have with their teachers, peers, and other school staff (Ma 2003). In addition to the effects of belonging to other social groups, SoSB provides academic and psychological benefits to individuals (Arslan et al. 2020; Tian et al. 2016). SoSB serves to create a school climate in which students feel safe (Ma 2003), to make students feel like important and respected members of the school (Booker 2004), and to create a healthy school.

Research shows that students feel they belong to a school when teachers create a safe educational environment (Anderman 2003), build good relationships with students (Allen et al. 2022; Sánchez et al. 2005), encourage mastery and learning rather than competition and performance (Stevens et al. 2007), value students, treat students fairly, provide academic and social support, and care about students (Allen et al. 2018, 2021b). Families can help students’ SoSB by providing emotional support (Steinberg and Morris 2001), being sensitive and tolerant, and establishing closeness (Allen et al. 2018). School principals can increase SoSB by providing extracurricular activities at school (Fredricks et al. 2004; Libbey 2004; Ma 2003; Whitlock 2006). Socializing and goal-orientation are beneficial for students to feel an SoSB and to support peer belonging (Allen et al. 2017, 2021a).

Studies which show the effects of SoSB on students’ academic development (Allen et al. 2018; Baumeister and Leary 1995; Sánchez et al. 2005), psychological well-being, and school health (Goodenow and Grady 1993; Ma 2003; Sánchez et al. 2005) have led policymakers and researchers to develop interventions to help students feel that they belong to their school. Individual and environmental factors come to the fore when examining the factors that can promote or hinder SoSB. Individual factors include “emotional intelligence (EI)”, academic motivation, mental health, achievement levels, and demographic factors, while environmental factors include relationships with teachers, parental support, peer support, norms, classroom climate, and school location (Allen et al. 2018; García-Moya et al. 2019; Korpershoek et al. 2020). Students’ perception of school as beneficial and the development of positive attitudes are individual factors that influence school belonging (Gillen O’Neel and Fuligni 2013; Van Ryzin et al. 2009).

Evidence suggests that both emotional and social factors jointly influence children’s positive school outcomes (e.g., Quílez-Robres et al. 2021). This underscores the importance of evaluating both EI and the social environment in relation to school belonging. As indicated by the research examples above, students’ SoSB is shaped by the attitudes and behaviors of multiple stakeholders, including the students themselves, teachers, school principals, and families. Consequently, it should be examined holistically, considering both direct and indirect effects. This highlights the need to address school belonging in conjunction with students’ EI and their socio-ecological environment.

## 2. Theoretical Context

The current study is based on two main theories: EI Theory and Ecological Systems Theory. Theories focused on EI differ in terms of conceptual and measurement assumptions and may be difficult to be generalized across different contexts. This study employs Mayer and Salovey’s (1997) Ability Model, which considers EI as a cognitive ability based on measurable mental processes rather than a set of personality traits. Mayer and Salovey define these specific abilities as the ability to perceive emotions, access emotions, and recognize emotions. This model provides a strong framework for explaining the relationship between students’ ability to process emotional information and regulate their social relationships in order to adapt to the school environment. It provides a suitable theoretical basis for tracking students’ development at the personal and social levels, as it emphasizes the developmental nature of emotional abilities. Several studies have shown that higher EI levels are associated with higher levels of subjective well-being (Sánchez-Álvarez et al. 2015), life satisfaction (Extremera and Fernández-Berrocal 2005), and better mental health (Ruiz-Aranda et al. 2012) and academic achievement (Costa and Faria 2015; MacCann et al. 2020). There are studies proving that EI is related to social relationship satisfaction, establishing relationships with friends and parents, and social acceptance (Brackett et al. 2004; Kokkinos and Kipritsi 2012; Lopes et al. 2003, 2004). Students with high emotional competence can develop behaviors such as cooperation, empathy, conflict resolution, and thus have a positive SoSB (Celume and Zenasni 2024; Moeller et al. 2020). Furthermore, meta-analytic studies show that emotional competencies have a positive effect on students’ school engagement and participation (e.g., Mavroveli and Sánchez-Ruiz 2011).

The second theory, Ecological Systems Theory, provides a functional framework for students’ school belonging by considering individual development in multi-layered environmental contexts. The theory, as theorized by Bronfenbrenner (1979), consists of a series of nested contextual environments, with the learner at the center and an emphasis on their synergistic relationships (Navarro et al. 2022). The diagram has four layers surrounding the learner at the center. The layers are microsystem, mesosystem, exosystem, and macrosystem. Some of the research on SoSB has undertaken the important task of providing a holistic framework of belonging based on a socio-ecological approach (Allen et al. 2016, 2022; Akbaba Altun and Turan Bora 2024). Previous studies examining school belonging have suggested that belonging is determined by students’ multiple experiences and interactions (Allen et al. 2018; Arslan et al. 2020; Akbaba Altun and Turan Bora 2024). The socio-ecological systems theory has attracted the attention of belonging researchers as it provides a framework that allows us to view these interactions and experiences holistically. Allen et al. (2016) proposed the Socio-Ecological School Belonging Model to investigate the factors affecting belonging in educational settings. Similarly, Akbaba Altun and Turan Bora (2024) examined school belonging from a socio-ecological perspective through the viewpoint of students. The socio-ecological systems theory is an appropriate model in terms of providing an opportunity to examine the effects of different stakeholders, institutions, individuals, etc. on belonging at each layer from the student to the outside world.

According to Weissberg et al. (2015), social/emotional learning processes depend not only on individual skills but also on the school environments that nurture and reinforce these skills. Bronfenbrenner’s framework offers a comprehensive analytical tool for examining the interaction between students’ emotional competencies and their environmental contexts, providing a theoretical basis for understanding belonging as the product of ongoing exchanges between the individual and the environment. A student’s ability to recognize and regulate their own emotions, as well as to understand others’ emotions, influences both their personal experiences and the relationships they form with environmental actors, such as family, teachers, and principals. The dynamic intersection of this multi-level structure with emotional competencies enhances the explanatory power of the ecological model, illustrating that belonging is woven not only structurally but also emotionally (Reschly and Christenson 2012; Pekrun 2014). The socio-ecological model seeks to explain the interplay between individuals and their surrounding ecosystems (Guo et al. 2018). EI refers to the ability to recognize and control one’s emotions in a way that reduces stress and manages one’s life, as well as being sensitive to the emotions of others and behaving appropriately in general (Ebersöhn and Eloff 2006). Molakeng et al. (2021) have incorporated EI as an internal resilience factor embedded within the socio-ecological model. Emotional competencies thus operate not only as individual traits but also as cross-ecosystemic factors; enhancing self-perception at the micro level while strengthening social bonds at the meso and macro levels. In this context, the present study aims to conceptualize the multi-layered nature of belonging and to provide a current and comprehensive framework for how emotional competencies interact with socio-ecological structures.

### Turkish Context

Türkiye has a collectivist, vertical hierarchical cultural structure with high power distance (Hofstede 2011). School belonging is sensitive to cultural, environmental, and geographical contexts and experiences (Allen et al. 2021c). High power distance and a collectivist cultural structure can affect social relationships that shape students’ school belonging and the bond they form with teacher authority (Cortina et al. 2017). On the other hand, frequent and comprehensive changes in education policies and practices in Türkiye can affect student belonging. Sudden changes in the curriculum and administrative practices can create feelings of instability and uncertainty among students. For example, the Ministry of National Education’s (MoNE) new curriculum model change announced in 2024 includes comprehensive transformations and has been rapidly implemented in all schools since 2025 (MoNE 2024b). School attendance and dropout problems are a concern for Turkish education policymakers. According to the MoNE’s 2022–2023 Monitoring and Evaluation Report, more than 15% of high school students were absent for more than 10 days in a month (MoNE 2024a). Furthermore, the enrolment rate of 15- to 19-year-olds in Türkiye is 69%, well below the OECD average (83%) (OECD 2022). The low level of SoSB in the world and in Türkiye is striking. The higher the level of education, the lower the level of SoSB. Given that the literature suggests that a lack of SoSB can lead to early school leaving (Goodenow 1993; Sithaldeen et al. 2022), this rate is worrying. The primary objective of this study is to examine the behaviors that school stakeholders should adopt to improve students’ SoSB based on teachers’ views. In line with this objective, the research questions are as follows:

RQ1: What should students do to improve their SoSB?
RQ2: What should teachers do to improve students‘ SoSB?
RQ3: What should families do to improve students‘ SoSB?
RQ4: What should guidance counselors do to improve students’ SoSB?
RQ5: What should school principals do to improve students’ SoSB?
RQ6: What should society do to improve students‘ SoSB?

Studies support the critical role of teacher/student relationships in fostering students‘ sense of school belonging (Cai et al. 2023; Crouch et al. 2014). Moreover, studies support the idea that teachers’ perceptions of student belonging align closely with students’ own perceptions (e.g., Crouch et al. 2014). Obtaining teachers‘ views on strategies to enhance students’ sense of belonging is therefore valuable for revealing teachers’ awareness and perspectives on this important construct. Given teachers’ significant influence on students’ academic, emotional, and social development, it was granted that they would provide high-quality, in-depth insights into students’ school belonging—an assumption that informed their selection as study participants.

## 3. Methods

This research was designed as basic qualitative research, also referred to as general or interpretive qualitative research, aimed at “understanding how people make sense of their lives and experiences” (Merriam and Tisdell 2016). This research design enables researchers to explore how participants perceive and interpret a given phenomenon (McMillan and Schumacher 2006; Merriam 2009).

### 3.1. Participants

The study group consists of 49 teachers selected through maximum diversity sampling. Participants represented 12 different provinces in Türkiye, varied in gender, and had a wide range of professional experience. Middle and high school teachers were specifically chosen, as students’ SoSB is typically lower at the secondary level than at the primary level. Among the participants, 37 were female and 12 were male, reflecting the broader gender distribution in Türkiye, where female teachers outnumber male teachers (MoNE 2022). Professional experience ranged from 1 year to over 21 years, with an average of 12 years. Participation was voluntary, and all teachers were informed that their responses would remain confidential. To protect privacy, each participant was assigned a pseudonym.

### 3.2. Procedure

The researchers developed seven open-ended questions aimed at examining the factors that could make students feel a sense of belonging to the school, in line with the purpose of this study. The questions were formulated based on two main theories (Intelligence Theory and Ecological Systems Theory) that form the background of this study, as well as the literature on school belonging. After the questions were prepared, two experts were first consulted. One of these experts was very experienced in qualitative research, while the other was an academic with research experience in school belonging. Following the expert review, three questions were revised. Next, pilot interviews were conducted with five teachers, and the questions were tested. After the pilot interviews, two questions were revised and rewritten. The seven open-ended questions, which were finalized by the researchers following the expert review and pilot interviews, were given to the teachers. After completing the questions, participants were provided with the researchers’ contact information for any future inquiries. To ensure objectivity, this study incorporated detailed documentation of each stage, enabling the tracking of the process from data collection to transformation, as well as secure storage of all research data. Efforts were made to ensure compatibility between the research questions and the research design, coding checks were carried out repeatedly by the researchers, and the roles of the researchers in the research process were explained. Credibility was supported by ensuring voluntary participation, presenting findings and results transparently, and quoting the participants’ statements to illustrate the codes. Participant transcripts were systematically compared with the codes that emerged.

### 3.3. Data Analysis

Analysis was initiated after the interview transcripts were created. Content analysis, which consists of coding, finding categories, and displaying data according to categories and codes, was used to analyze the data. According to Patton (2014), content analysis is any qualitative data reduction and interpretation effort to identify underlying consistencies and meanings by taking qualitative material. Content analysis means that the text is searched for recurring words or themes. In this study, descriptive coding—the statement summarizing the main topic of the quotation—and in vivo coding—the statement taken directly from the participant’s words (Saldaña 2021)—were used, while the codes were revealed by the researchers. The first author read the data once from beginning to end before coding. A second reading was then undertaken and raw codes were extracted. The raw codes were shared with the second author. The second author read the raw data and raw codes, compared them and made corrections, deletions, and additions. The authors reviewed and finalized these codes together. The codes were categorized by the first author and the themes were reorganized with the second author.

## 4. Results

Teachers‘ responses to the questions were analyzed through content analysis, and the roles of students, families, teachers, guidance counselors, school principals, and society in terms of students’ SoSB were identified separately. They are presented in Table 1 in terms of categories and codes.

### 4.1. How Do Students Improve Their SoSB?

The things students should do to feel an SoSB were grouped under the categories of “awareness” and “active participation”. In order for students to feel that they belong to their school, they should first have a positive attitude toward the school and should be aware of its importance. Teachers believe that a positive attitude toward the school is related to trust in the school, love for the school, perception of the school as home, and respect for the school.

Ayşe: *Students should see the school as a family, they should know and understand that all the elements there are as important and valuable as themselves, and they should act accordingly.*

Zeynep: *Students should know that their dreams can be realized with school and education. Students should love school. They should trust the school, their friends, teachers, and principals.*

Teachers emphasized that students need to be active in order to feel that they belong to the school. It was stated that in order for students to be active, they need to follow school rules, communicate well with their teachers and friends, adapt to the school, use school facilities, participate in school activities, take responsibility in these activities, and make an effort.

Rüya: *They should benefit from the opportunities offered by the school, should be aware of their responsibilities and strive to improve themselves in every way.*

Ahmet: *They should communicate with their teachers and friends, not be prejudiced and try to be part of the process.*

### 4.2. What Teachers Should Do to Improve Students’ SoSB

The actions teachers should take to improve students’ SoSB were categorized as “academic support” and “emotional support”. To help students feel a sense of belonging to their school, teachers should encourage their academic curiosity, involve them in classroom activities, provide guidance, be fair in their behavior, conduct student-centred lessons, motivate them, and value individual differences.

Saliha: *Teachers have a lot of work to do in this regard, because although many students actually like to come to school, they do not like the lessons and enjoy the breaks and free time more. Instead, they can make lessons more enjoyable and develop the children’s SoSB by creating a sense of curiosity in the children, by being constantly energetic and not reflecting this back to the class, even when they are experiencing something emotionally negative…*

Teachers establishing connections with students, showing them love, closeness, compassion, and interest, getting to know them and understanding their ideas, respecting them, making them feel valued, and supporting them will help students’ SoSB.

Mehmet: *They should show their love to the students. When the student feels the love of the teacher, he/she thinks that he/she belongs to the school.*

Elif: *Teachers should endear themselves to their students, should not focus only on academic achievement, and should make each student feel special.*

### 4.3. What Families Should Do to Improve Students’ SoSB

The things the family should do to help students’ SoSB were grouped under the categories of “student-centred behaviours” and “school-centred behaviours”. For students with SoSB, families need to be aware of their children, know their abilities, differences, and interests, monitor them, avoid comparing them with their peers or friends, and make them feel valued.

Arda: *Families should have a happy time with their children. They should increase their communication with them and make them understand their real problems. They should understand the emotional changes caused by adolescence and be more relaxed about the ups and downs in their behaviour.*

Murat: *They should live in the same world in the same house. They should organise family activities and be able to see eye to eye with their children and stay together.*

Teachers have stated that parents’ cooperation with the school, their positive attitude toward the school, their encouragement of their children to attend school, their support for the school, and their communication with the school will help their children feel that they belong to the school.

Betül: *Such families should at least accept that they should cooperate with the school guidance counsellor and the student’s teachers and act in accordance with guidance.*

Selim: *They should not denigrate the school and the teacher in front of their children. They should explain the importance of school and follow up with their students.*

### 4.4. What Guidance Counselors Should Do to Improve Students’ SoSB

The things the guidance counselor should do to make students feel that they belong to the school were grouped under the headings of “school-wide activities” and “psychological counselling”. The guidance counselor’s efforts to educate parents, support them during and after the training, work together with families, teachers, and school principals, and communicate with families, students, and teachers can improve students’ SoSB.

Merve: *You should interview the students individually or in groups in a certain order, not only with the problematic students.*

Zeynep: *They can use student councils more effectively. They can create and develop SoSB by counseling and guiding not only the students with problems, but also every student in the school at certain intervals, using techniques related to their field.*

Teachers also emphasized that if psychological counselors provide counseling based on a relationship of trust, and if they are familiar with their students and support them, students will feel a sense of belonging to the school.

Deniz: *They should ensure that children feel comfortable communicating with them. They should have confidence. Students should feel the need to tell the guidance counsellor when they encounter difficulties or experience something positive.*

Faruk: *If the student has adjustment problems due to the overprotective attitude of the family, it is important to gradually reduce the number of parents coming to school with the student and to ensure that the student gets used to his friends and class. If the student has difficulties in getting used to the teacher and his friends (personal characteristics of the student), different games can be played to emphasise that the school is interesting and nice and to get the students to know each other better.*

### 4.5. What School Principals Should Do to Improve Students’ SoSB

Teachers’ views on what school principals should do to make students feel a part of the school were grouped under the categories of “administrative behaviour” and “interpersonal behaviour”. Teachers emphasized that if school principals organize social, cultural, and sporting activities in their schools, cooperate with stakeholders, prepare an adaptation program, establish a democratic school system, improve the physical and social environment of the school, and increase peace in the school, students can feel a sense of belonging.

Asya: *School principals should understand child psychology according to their level, organise the school accordingly and have strong communication with parents and students. They should involve students in the process by organising projects and activities, they should not always sit in their rooms, they should be in contact with students.*

Songül: *By making the physical conditions of the educational environment as comfortable and functional as possible within the framework of its financial power, such as society support, perhaps with external support, and by providing equal conditions not only in school but also in out-of-school cultural activities and regardless of the financial power of the student, it can improve the students’ SoSB.*

Teachers expressed the following views: if school principals show empathy toward students, communicate effectively with parents and students, and give students confidence, students can feel a sense of belonging.

Rıza: *I think the most important thing that school principals should be concerned about and improve upon when they realise their shortcomings is to have a loving, compassionate, sympathetic and positive attitude towards their students, as well as a balanced, respectful, determined and sincere attitude. It is not possible for a headteacher who is anxious, grumpy and moody to improve students’ SoSB.*

### 4.6. What Society Should Do to Improve Students’ SoSB

The opinions of the teachers were divided into two categories: “awareness” and “participation”. Teachers expressed the following views on the fact that society’s awareness of students and the importance of school and raising awareness will make students feel that they belong to their school.

Selim: *In order to make the students feel that they belong to the school, the society should be very aware of the period in which the students are growing up, they should know the interests, wishes and expectations of the students very well. They should seek answers to questions such as what young people want and expect, and empathise with them.*

If society is active in guiding the school, making them love school, supporting education, cooperating with the school, sharing experiences, and participating in school activities, students will feel that they belong to the school.

Tarık: *The society can make the students feel that they belong to the school by supporting the school’s activities that improve the quality of education or sports (by providing financial support for a sports team or an individual branch)art (painting or art workshop).*

Ceren: *Society and the environment should do what is necessary and cooperate with the school to make the child feel belong to the school.*

### 4.7. A Holistic View of Students’ SoSB

Additionally, teachers stated in the following statements that students’ SoSB should be considered holistically, that stakeholders should work together in cooperation, that school principals and teachers should also feel that they belong to the school, and that it is necessary to create such a school culture.

Seçil: *The climate created in the school is reflected in all members of the school. Therefore … trust is very important. People are peaceful in a safe environment and feel a sense of belonging. Therefore, the harmony between the teacher and the school principals will have a positive effect on the students.*

Özgür: *A student’s SoSB is not a situation that can be ensured only in the context of the student, the society, the student, the teacher or the family. The issue should be addressed with a holistic understanding. The absence of one element should be compensated by the other.*

**Figure 1:**
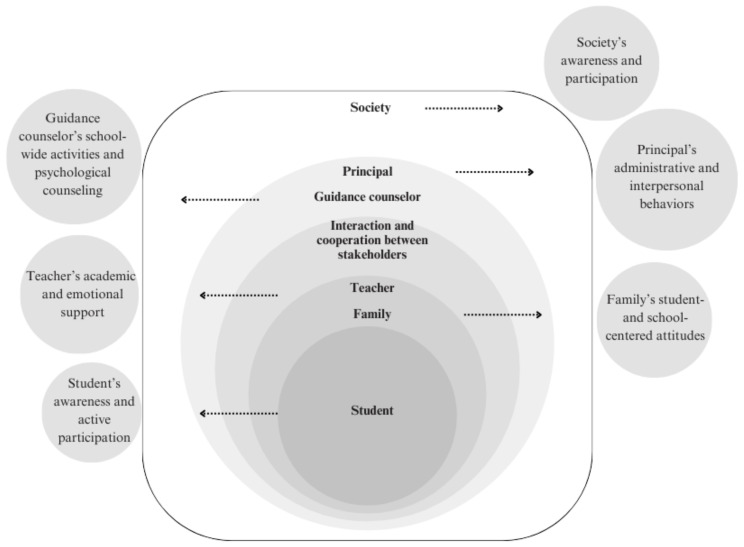
A teacher perspective on the framework of SoSB.

The roles of the stakeholders that will be effective in making students feel that they belong to the school were discussed according to the socio-ecological approach and are summarized in Figure 1.

## 5. Discussion

This study adopts a holistic approach to students’ SoSB by examining the combined influence of EI and social environment factors—in students’ SoSB. Drawing on the ecological approach to human development, we developed a framework outlining stakeholders’ responsibilities based on teachers’ views. EI, which encompasses skills such as empathy, emotional regulation, and interpersonal awareness, aligns closely with the relational dimensions embedded in each layer of the socio-ecological model. Within this framework, students’ emotional competencies are both shaped by and contribute to the interactions they experience across the microsystem (e.g., family and teachers), mesosystem (e.g., home-school connections), exosystem (e.g., school leadership), and macrosystem (e.g., society values). In this way, EI is not merely an internal capacity but a relational force that mediates students’ belonging within their socio-ecological environments. By critically interweaving these perspectives, the present study offers a holistic and context-sensitive account of the factors that foster students’ SoSB.

Students are at the center of the socio-ecological framework of belonging. In this layer, individual-level characteristics that influence students’ SoSB come to the fore. The themes that emerged in our study in this layer are awareness and active participation. Researchers have described students’ perception of school as useful and developing positive attitudes toward school belonging (Gillen O’Neel and Fuligni 2013; Van Ryzin et al. 2009). As posited by Allen et al. (2016), students’ individual characteristics that support SoSB are categorized as follows: emotional condition, academic motivation, and personal characteristics. According to Salovey and Mayer (1990), high EI enables individuals to think clearly while supporting intuition and insight. Analysis of teachers‘ views on the positive effect of high awareness on students’ SoSB suggests that such awareness is closely linked to students’ EI. Demetriou et al. (2023) also revealed the relationship between students’ awareness and intelligence. In the current study, “active participation” also emerged as a behavior that supports students’ school belonging. Students’ behaviors such as cooperation and conflict resolution are related to their emotional competencies (Celume and Zenasni 2024), and prior research has found that social acceptance is also related to EI (Brackett et al. 2004; Kokkinos and Kipritsi 2012; Lopes et al. 2003, 2004). Based on the literature, students’ active participation behavior in school can be considered as a result of their EI (Mavroveli and Sánchez-Ruiz 2011).

The microsystem level of the socio-ecological framework includes the family and the school with which the student interacts one by one. The roles expected of teachers are divided into two themes: academic and emotional. The most effective level for SoSB, together with the individual level, is the microsystem level. The place of teachers at this level in SoSB has been extensively researched. According to Celume and Zenasni (2024) teachers can contribute to the creation of more inclusive and supportive learning environments by emphasizing the development of well-rounded competencies in the classroom, teachers can foster a sense of belonging and society by empowering students with emotional competencies and interpersonal skills. There is substantial evidence that teachers’ behavior and support have a greater impact on students’ academic and social development than other factors (Anderman 2003; Brewster and Bowen 2004; Hattie 2009; Sakiz 2012). Among the studies that align with our findings, teacher fairness behaviors are consistent with Sakiz’s (2012) results, bonding behaviors is consistent with Uslu and Gizir’s (2017) study, and effective communication is consistent with Sánchez et al.’s (2005) research findings.

Within the same layer, the family—another stakeholder in the student’s immediate environment—was assigned two roles: student-oriented and school-oriented. Student-oriented roles include getting to know the student, showing interest, and making the student feel valued. School-oriented roles involve cooperating with the school, having a positive attitude toward school, directing the student to school, and supporting the school. Previous research has shown students experience a stronger sense of belonging when they receive support, attention, and affection from parents (Brewster and Bowen 2004; Steinberg and Morris 2001; Wang and Eccles 2012). Similarly, Allen et al. (2018) stated that closeness and tolerance from family can improve students’ sense of belonging.

The mesosystem layer is the layer of relationships that expresses the results of the student’s interaction with the teacher, family, peer group, etc. Guidance counselors’ practices are both related to the mesosystem and indirectly related to the microsystem. The roles of guidance counselors of SoSB are divided into two themes: school-wide and psychological counselling activities. Perceiving guidance counselors as trustworthy advisors will reassure students, strengthen their belief that their problems can be resolved within the school, and reduce the likelihood of looking for solutions outside of school. Furthermore, previous studies have shown that providing a caring and supportive educational environment is one of the key factors in fostering students’ SoSB (Baumeister and Leary 1995; Fredricks et al. 2004; Libbey 2004; Ma 2003).

The leadership of school principals, their legislation-oriented bureaucratic duties, and their influence on school culture and climate enable them to take part in the exosystem layer, which is a more general layer that constitutes the student environment. When we look at the findings related to the roles of school principals, we see activity, cooperation, and administrative skills in the roles under the managerial theme. Under the humanitarian theme, communication and trust were found. Extracurricular activities have also been found in various studies (Fredricks et al. 2004; Libbey 2004; Ma 2003; Whitlock 2006). Soria et al. (2013) studied 1865 students who participated in school opening events and reported that these students felt a greater SoSB than those who did not participate. It has also been shown that school principals can influence SoSB through school policies and practices (Allen et al. 2016). The reason why teachers emphasize the importance of interpersonal behavior of school principals for student school belonging can be interpreted in light of the high power distance and vertically hierarchical culture structure which can hinder direct student/principal relations. In addition, various researchers have emphasized that students’ sense of safety at school positively influences their SoSB (Libbey 2004; Whitlock 2006). To mitigate the negative effects of uncertainty and rapid change on belonging, it may be effective for school principals to foster an empathetic, democratic, and collaborative school environment.

In the macrosystem layer, the categories of behavior expected from society are awareness and participation. Research findings highlighting the need to consult society members in the development of school policies are consistent with our study (Whitlock 2006). Other opinions of the participants emphasized stakeholder cooperation, the sense of belonging among school principals and teachers, and the role of school culture. A strong sense of belonging among both staff and students, coupled with positive school culture, can further support students’ SoSB.

## 6. Conclusions, Implications, and Limitations

This study examined the behaviors that school stakeholders should adopt to improve students’ SoSB according to EI theory and the socio-ecological framework, based on teachers’ views. This study examined student belonging within the context of both emotional and social environment, emphasizing that such a multidimensional phenomenon cannot be explained solely by student behaviors. It highlights the importance of the roles played by all stakeholders, from those in closest contact with the student to those with more indirect involvement. In the ecosystem in which the student is located, each unit should have responsibilities according to the unitary/separate approach. According to the holistic and interactive perspective, each unit should interact with one another and the SoSB of the student should be provided with a holistic approach. It was emphasized that EI is an important factor in the formation of belonging and that the student’s emotional competencies should be supported.

In the context of the results of this research, some suggestions can be offered to policymakers and practitioners. First of all, the responsibility of stakeholders in students’ school belonging can be included in both school principal and teacher training programs, family trainings and seminars, and society cooperation activities. Efforts to raise awareness of the role of all stakeholders in ensuring students’ belonging can support the development of every student in the school and support equality policies. It may be useful to motivate students to participate in non-academic cooperative activities with teachers and school principals. On the other hand, it can be ensured that the development of EI of students, teachers, school principals, families, and society members can be supported. Integration of EI into activities and workshops involving adults should be supported in the school.

This study has some limitations that should be addressed in future studies. Firstly, it would have been stronger if the views of school principals and counselors as well as teachers were included in the study group. Quantitative and longitudinal studies examining students’ perceptions and views of school belonging could reduce the limitations of this study, which is cross-sectional and qualitative. On the other hand, the policies and practices of the Ministry, provincial and district education organizations at the macro level should also be evaluated. Other limitations of this study relate to theories. Talent-based EI evaluations can show a high correlation with personality traits, raising concerns about construct redundancy. Furthermore, although Bronfenbrenner’s socio-ecological model allows for a layered analysis of the relationships between individuals and their environment, it remains descriptive and may have limited explanatory power regarding how emotional processes shape environmental interactions. In future studies, alternative or complementary theories that provide further explanation for understanding belonging may be considered.

## Figures and Tables

**Table 1:** Categories and codes.

	Category 1 and Codes	Category 2 and Codes
Student	Awareness Positive attitude toward school (*n* = 15) Awareness of the importance of the school (*n* = 12)	Active participation Rule compliance (*n* = 5) Communication (*n* = 8) Adaptation (*n* = 3) Use of school facilities (*n* = 4) Participation (*n* = 8) Responsibility (*n* = 10) Effort (*n* = 3)
Teacher	Academic support Curiosity (*n* = 3) Activity-based instruction (*n* = 11) Guidance (*n* = 8) Fairness (*n* = 6) Student-centred education (*n* = 4) Motivation (*n* = 5) Attention to individual differences (*n* = 6)	Emotional support Connecting (*n* = 19) Familiarity with students (*n* = 10) Respect (*n* = 5) Value affirmation (*n* = 6) Support (*n* = 9) Communication (*n* = 10)
Family	Student-centred behaviors Familiarity with children (*n* = 7) Concern (*n* = 6) Monitoring (*n* = 3) Avoidance of comparison (*n* = 3) Value affirmation (*n* = 5)	School-centred behaviors School collaboration (*n* = 8) Positive attitude toward school (*n* = 12) Student orientation to school (*n* = 10) Support for school (*n* = 11) Communication (*n* = 7)
School principal	Administrative behaviors Activity management (*n* = 28) Democratic school structure (*n* = 14) School environment improvement (*n* = 10) Collaboration (*n* = 5)	Interpersonal behaviors Empathy (*n* = 13) Communication (*n* = 8) Confidence building (*n* = 6)
Guidance counselor	School-wide activities Parent training (*n* = 7) Collaboration (*n* = 15) Communication (*n* = 18)	Psychological counseling activities Familiarity with children (*n* = 7) Trust-based relationship (*n* = 3) Support (*n* = 14)
Society	Awareness Empathy (*n* = 7) Awareness of the importance of school (*n* = 10) Awareness raising (*n* = 7)	Participation Educational support (*n* = 12) Promotion of school appreciation (*n* = 10) School collaboration (*n* = 3)

## Data Availability

The data presented in this study are available on request from the corresponding author. The data are not publicly available due to privacy.
